# Localization of Canine Brachycephaly Using an Across Breed Mapping Approach

**DOI:** 10.1371/journal.pone.0009632

**Published:** 2010-03-10

**Authors:** Danika Bannasch, Amy Young, Jeffrey Myers, Katarina Truvé, Peter Dickinson, Jeffrey Gregg, Ryan Davis, Eric Bongcam-Rudloff, Matthew T. Webster, Kerstin Lindblad-Toh, Niels Pedersen

**Affiliations:** 1 Department of Population Health and Reproduction, School of Veterinary Medicine, University of California Davis, Davis, California, United States of America; 2 Biomedical Centre, Department of Animal Breeding and Genetics, Swedish University of Agricultural Sciences, Uppsala, Sweden; 3 Department of Surgical and Radiological Sciences, School of Veterinary Medicine, University of California Davis, Davis, California, United States of America; 4 Department of Pathology, School of Medicine, University of California Davis, Davis, California, United States of America; 5 Department of Medical Biochemistry and Microbiology, Uppsala University, Uppsala, Sweden; 6 Broad Institute of Harvard and Massachusetts Institute of Technology (MIT), Cambridge, Massachusetts, United States of America; 7 Department of Medicine and Epidemiology, School of Veterinary Medicine, University of California Davis, Davis, California, United States of America; Institut de Genetique et Microbiologie, France

## Abstract

The domestic dog, *Canis familiaris,* exhibits profound phenotypic diversity and is an ideal model organism for the genetic dissection of simple and complex traits. However, some of the most interesting phenotypes are fixed in particular breeds and are therefore less tractable to genetic analysis using classical segregation-based mapping approaches. We implemented an across breed mapping approach using a moderately dense SNP array, a low number of animals and breeds carefully selected for the phenotypes of interest to identify genetic variants responsible for breed-defining characteristics. Using a modest number of affected (10–30) and control (20–60) samples from multiple breeds, the correct chromosomal assignment was identified in a proof of concept experiment using three previously defined loci; hyperuricosuria, white spotting and chondrodysplasia. Genome-wide association was performed in a similar manner for one of the most striking morphological traits in dogs: brachycephalic head type. Although candidate gene approaches based on comparable phenotypes in mice and humans have been utilized for this trait, the causative gene has remained elusive using this method. Samples from nine affected breeds and thirteen control breeds identified strong genome-wide associations for brachycephalic head type on Cfa 1. Two independent datasets identified the same genomic region. Levels of relative heterozygosity in the associated region indicate that it has been subjected to a selective sweep, consistent with it being a breed defining morphological characteristic. Genotyping additional dogs in the region confirmed the association. To date, the genetic structure of dog breeds has primarily been exploited for genome wide association for segregating traits. These results demonstrate that non-segregating traits under strong selection are equally tractable to genetic analysis using small sample numbers.

## Introduction

**Figure 1:**
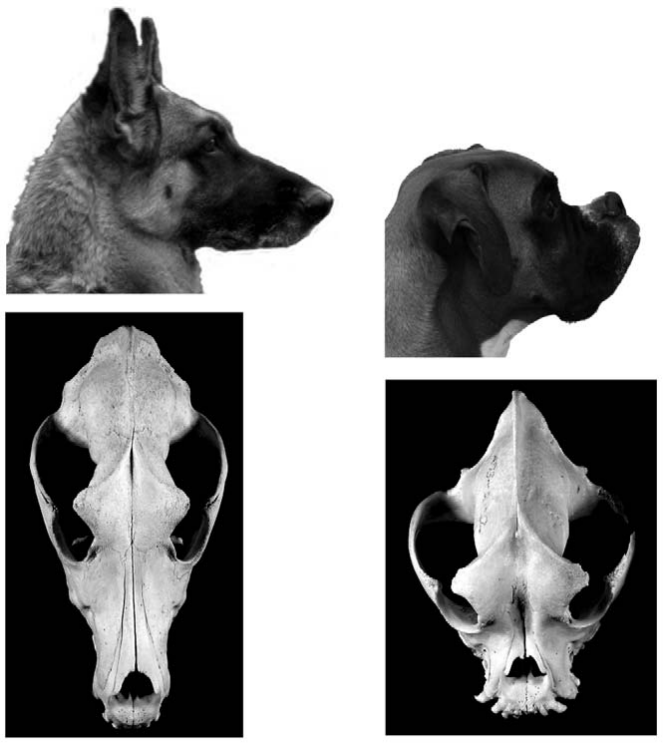
Brachycephaly in dogs. Comparison of photographs (Photos Mary Bloom, courtesy of AKC) and skulls from a German Shepherd Dog with a wild-type skull shape (non-brachycephalic) and a brachycephalic Boxer.

Each dog breed is defined by a specific combination of morphological, behavioral and coat color traits. Many of these phenotypic traits, as well as the underlying mutations, are shared between breeds. Some of these phenotypic traits have no effect on health, while others may be associated with certain medical problems. Brachycephaly (Figure 1) is one of the most easily recognizable phenotypic traits of the latter type and cause dramatic morphological changes in a substantial proportion of dog breeds.

Brachycephaly is characterized by severe shortening of the muzzle, and therefore the underlying bones, and a more modest shortening and widening of the skull [1]. Brachycephalic breeds such as the Boxer, Boston Terrier, Pekingese, and Bulldog exhibit prognathism and have wide-set, round eyes. Based on the history of many brachycephalic breeds, this phenotype was originally selected in dogs that were used for fighting, based on the idea that this head shape is more powerful for biting [2], [3]. Brachycephaly is associated with a number of medical conditions in the dog including breathing abnormalities, cleft palate and lip and, in some breeds, increased risk of gliomas [4], [5], [6], [7]. Despite these serious medical issues, dogs that exhibit brachycephalic head type have been favored for hundreds of years due to the similarity of their skull shape to that of human infants [8]. With the use of artificial insemination and Caesarean sections that allow some brachycephalic breeds to reproduce, selection for more extreme versions of this phenotype has occurred and it is likely that genetic modifiers exist for this trait [9], [10]. There is little variation within these breeds for brachycephaly, indicating that individuals are largely fixed for the causative allele. Based on crosses performed between breeds, brachycephaly is a semi-dominant trait [1]. Archeological evidence suggests that brachycephaly existed before the formation of modern breeds so it is likely that the major locus that confers this phenotype is common among affected breeds [11]. The gene responsible for brachycephaly is unknown. An association between a variant of the canine TCOF1 gene and brachycephaly has been suggested in the past [12] but recent experiments do not support those findings [9].

Phenotypic traits that are common across many breeds, such as brachycephaly, are likely to be identical by descent, just as many other specific genetic traits have been shown to be shared between different dog breeds [13], [14], [15], [16], [17], [18], [19], [20], [21], [22]. Most of this phenotypic sharing ceased around 100 years ago when breed standards were fixed and stud books closed. Before this time, certain phenotypic traits would appear in individual dogs and desirable traits would be incorporated into a proto-breed of the time. Strong selection for desirable phenotypic traits that bred true led to homozygosity within breeds [23].

This unique structure of dog breeds has been useful in determining the genetic basis for many desirable and deleterious traits, an effort facilitated by new genetic technologies emanating from the sequencing of the canine genome [24]. Whole genome association analysis studies that utilize single nucleotide polymorphism (SNP) markers have been used to identify the molecular causes of various traits and conditions including genetic mutations within breeds that cause coat color variations [15], hairlessness [25] and defects in spinal development [26]. Trait identification involves single breeds that segregate the trait of interest followed by fine structure mapping using additional breeds that segregate the trait [14]. This two stage mapping approach has been very successful and requires modest numbers of individuals. Precise mapping of major loci responsible for trait variation has been accomplished with relatively small numbers of dogs as compared to the large numbers of individuals required for trait identification in genetically diverse human populations. This same unique genetic structure of modern dog breeds can also be advantageous for genetic studies across various breeds. Across breed association for breed stereotypes has been performed for a number of polygenic morphological and behavioral traits using relatively few SNPs across 148 breeds. Multiple significant associations were obtained for many of the traits [27]. Recently, a large scale multibreed association analysis using 797 dogs from 72 breeds was used to define the likely mutation causing chondrodysplasia in dogs [28].

In this study, an across breed mapping approach using SNP arrays and fine mapping techniques was used to identify chromosomal locations that are associated with brachycephaly using comparable sample numbers to studies that have investigated traits within single breeds. This approach takes advantage of the allelic nature of traits between breeds and the extended regions of linkage disequilibrium within breeds as well as the power of strong artificial selection for breed-defining traits [24]. We propose that using SNP technologies to discern the molecular basis for traits and diseases can be successfully accomplished across multiple breeds as well as within single breeds and that this approach is especially useful for traits that are homozygous within breeds and have undergone strong selection as breed-defining characteristics. Using a modest number of samples, correct chromosomal locations were identified by genome-wide association for three positive control non-segregating traits. Brachycephalic head type was associated with multiple SNPs on Cfa1 using 20 cases and 31 controls. These results were confirmed in an independent dataset in which decreased relative heterozygosity along the chromosome also verified the position. Additional SNP genotyping in affected and unaffected dogs identified a region of overlap in homozygosity between affected breeds of 31 Kb.

## Results

**Figure 2:**
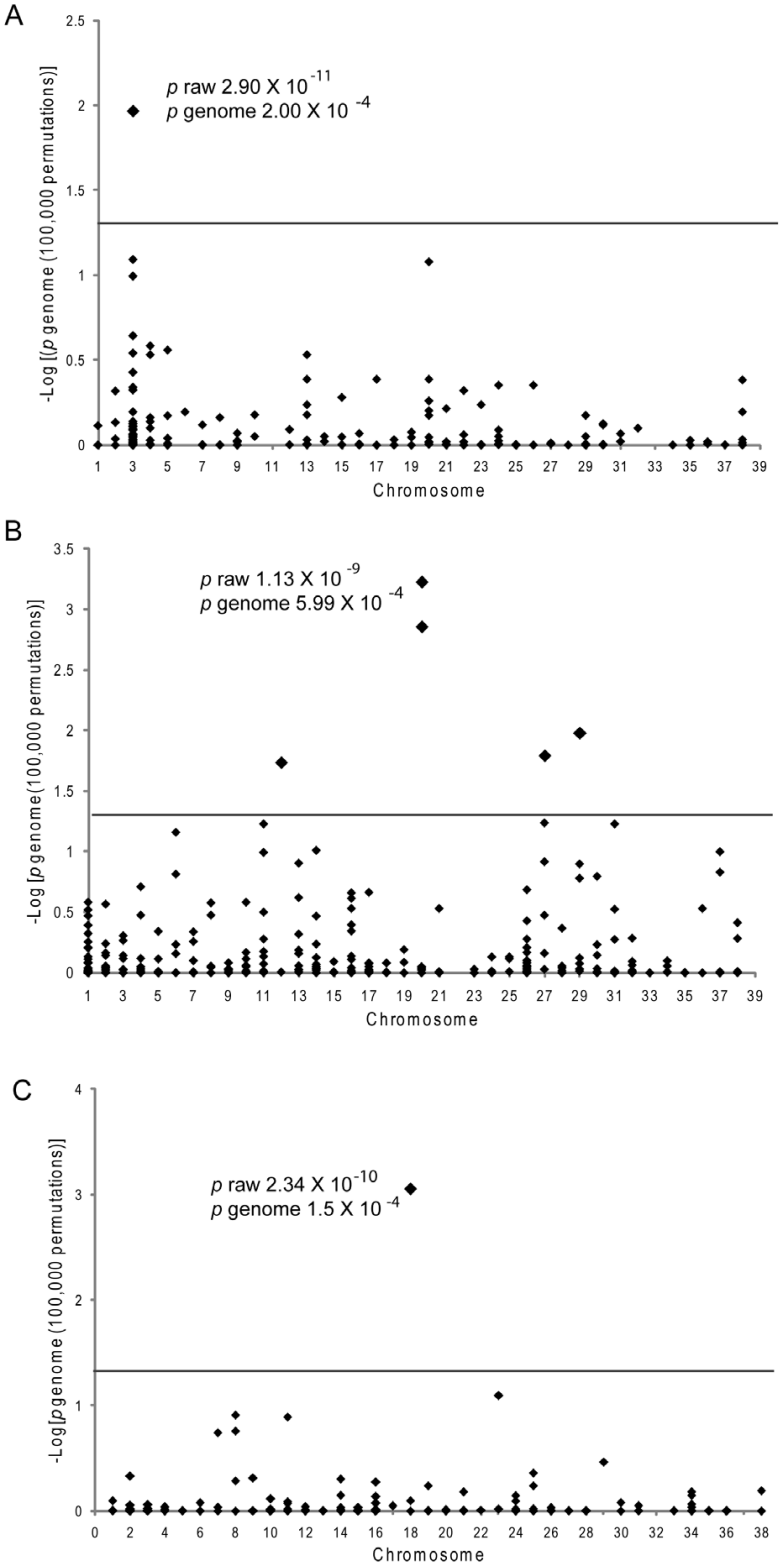
Across breed genome-wide association for positive control loci. A.-log 10 of the permuted (100,000) p values (y axis) is plotted by chromosome (x axis). The horizontal line indicates a p value of 0.05. The most significant associations have the raw and permuted p values indicated in the figure. A. Hyperuricosuria B. White spotting and C. Chondrodysplasia.

To demonstrate the power of across breed mapping, three positive control traits, hyperuricosuria, white spotting and chondrodysplasia, were selected for genome wide association analysis. Hyperuricosuria (Huu) is a change in urinary metabolism caused by a mutation in the SLC2A9 gene [14]. It is fixed in the Dalmatian breed and occurs at high allele frequency in the Bulldog and Black Russian Terrier breeds [29]. Using 10 total affected dogs from three breeds and 59 controls from 25 breeds, a genome wide association was performed. Strong association (χ^2^ test, nominal p value (*p*_raw_) = 2.89×10^−11^ and p value corrected by permutation testing (*p*_genome_) = 2.0×10^−4^) was detected on CFA 3 at 70.2 Mb adjacent to the causative gene (SLC2A9; 72.2–72.4 Mb) (Figure 2). The second highest association to a different chromosome was 100 times less likely than the correct one.

A second positive control trait, white spotting, is caused by mutations in the MITF gene and has been shown to be allelic between many different dog breeds [15]. Strong genome wide association was obtained using 31 cases for white spotting from 11 breeds and 31 controls from 14 breeds (χ^2^ test, nominal *p* value (*p*_raw_) = 1.31×10^−9^ and permutated p value corrected for genome-wide search (*p*_genome_) = 5.99×10^−4^) on CFA 20 at 24.88 Mb (MITF; 24.85–24.88 Mb) (Figure 2). Although the correct chromosome assignment and location had the most significant *p* value for white spotting, there were three other chromosomes that reached permuted *p* values of less than 0.05, however these were between 24–40 fold less likely than the correct chromosome.

The third positive control locus was canine chondrodysplasia, a dominant trait that is alleleic between many different dog breeds [1]. A large scale multibreed mapping approach recently identified the mutation as a retrotransposon insertion at 23.4 MB on CFA 18 (Parker et al. 2009). Chondrodysplasia was mapped using 18 cases (6 breeds and 3 crossbred dogs) and 27 controls (11 breeds and 4 cross bred dogs) to canine chromosome (CFA) 18 (χ^2^ test, nominal *p* value (*p*_raw_) = 2.34×10^−10^ and p value corrected for genome-wide search (*p*_genome_) = 1.5×10^−4^ on the basis of 100,000 permutations) (Figure 2). The association with the SNP (Chr18.23298242) on CFA 18 is 91 times more significant than for the next highest association in the genome.

In order to overcome the issue of breed-specific differences resulting in widespread false positives, several breeds were used that, aside from the trait under investigation, were phenotypically similar to the affected individuals. Affected breeds for brachycephalic head type included Japanese Chin, Affenpinscher, Pekingese, French Bulldog, Pug, Boston Terrier, Boxer, Bulldog and Shih Tzu. Control dogs were dolichocephalic (long muzzle) or mesaticephalic (medium muzzle length); they included Akitas, Bloodhounds, Dalmatians, Coonhounds, Belgian Tervurens, Whippets, Great Danes, German Shepherd Dogs and Black Russian Terriers. A complete list of affected and control dogs are given in supplemental Table S1 for the positive control loci as well as brachycephaly.

**Figure 3:**
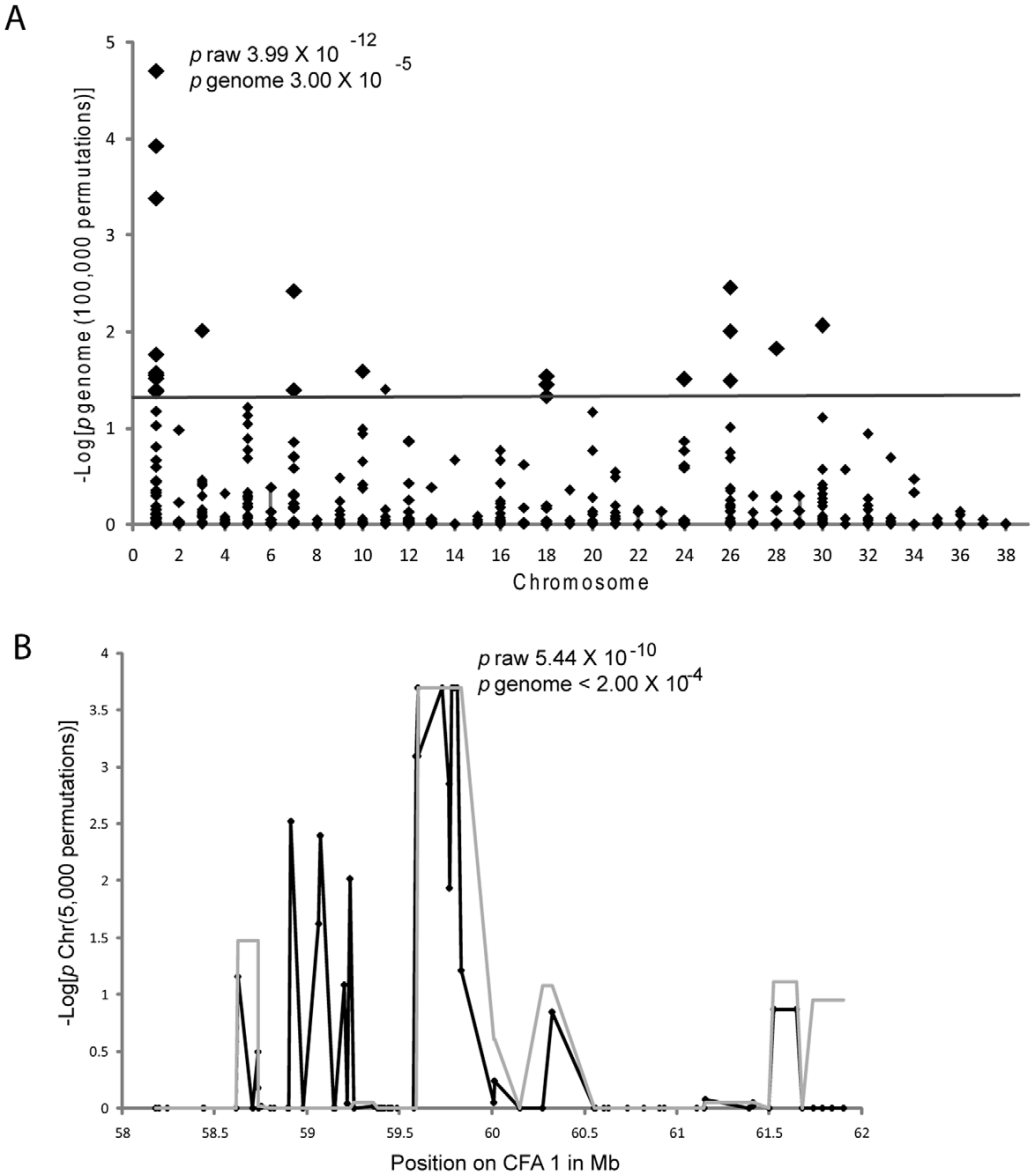
Across breed genome-wide association for brachycephaly. A. -log 10 of the permutated (100,000) p values (y axis) for genome wide association for brachycephaly across dog breeds are plotted by chromosome (x axis). Raw p values as well as the permutated p value for the most significant associations are shown near the peak. B. Chromosome-wide association for SNPs (black) and haplotypes (grey) defined by the four gamete rule were performed using Haploview using 50000 permutations. -log10 of permuted p values (y axis) are plotted against position on the chromosome in Mb (x axis).

Using this method, strong genome wide association was identified for multiple SNPs for brachycephalic head type (Figure 3). The brachycephalic head type locus mapped to a region on CFA 1 (Chr1∶59536208-Chr1∶59832965) (χ^2^ test, nominal *p* value (*p*_raw_) = 4.0×10^−12^ and *p* value corrected for genome-wide search (*p*_genome_) = 3.0×10^−5^ on the basis of 100,000 permutations). The genetically complex nature of the brachycephalic head type phenotype may be contributing to the associations across many different chromosomes; however, based on permutation testing, the association on CFA 1 is 1340 times stronger than the next highest association.

Based on the results obtained from the SNP arrays, haplotype analysis was performed for brachycephaly. Brachycephaly showed highly significant associations of single SNPs as well as three haplotypes spanning 296 kb (χ^2^ test, nominal p value (*p*_raw_) = 9.47×10^−13^ and *p* value corrected for genome-wide search (*p*_genome_)<2.0×10^−4^) (Figure 3).

In order to further confirm the chromosome assignment for brachycephaly, a second independent data set was used for genome wide association. This dataset had fewer affected and control breeds but more individuals from each breed (A complete list of dogs in the second dataset is available in supplemental Table S2). For brachycephalic head type, the same region on CFA 1 was identified with a raw *p* value of 5.287×10^−49^. The next most significant *p* value for brachycephaly was 2.44×10^−36^.

**Figure 4:**
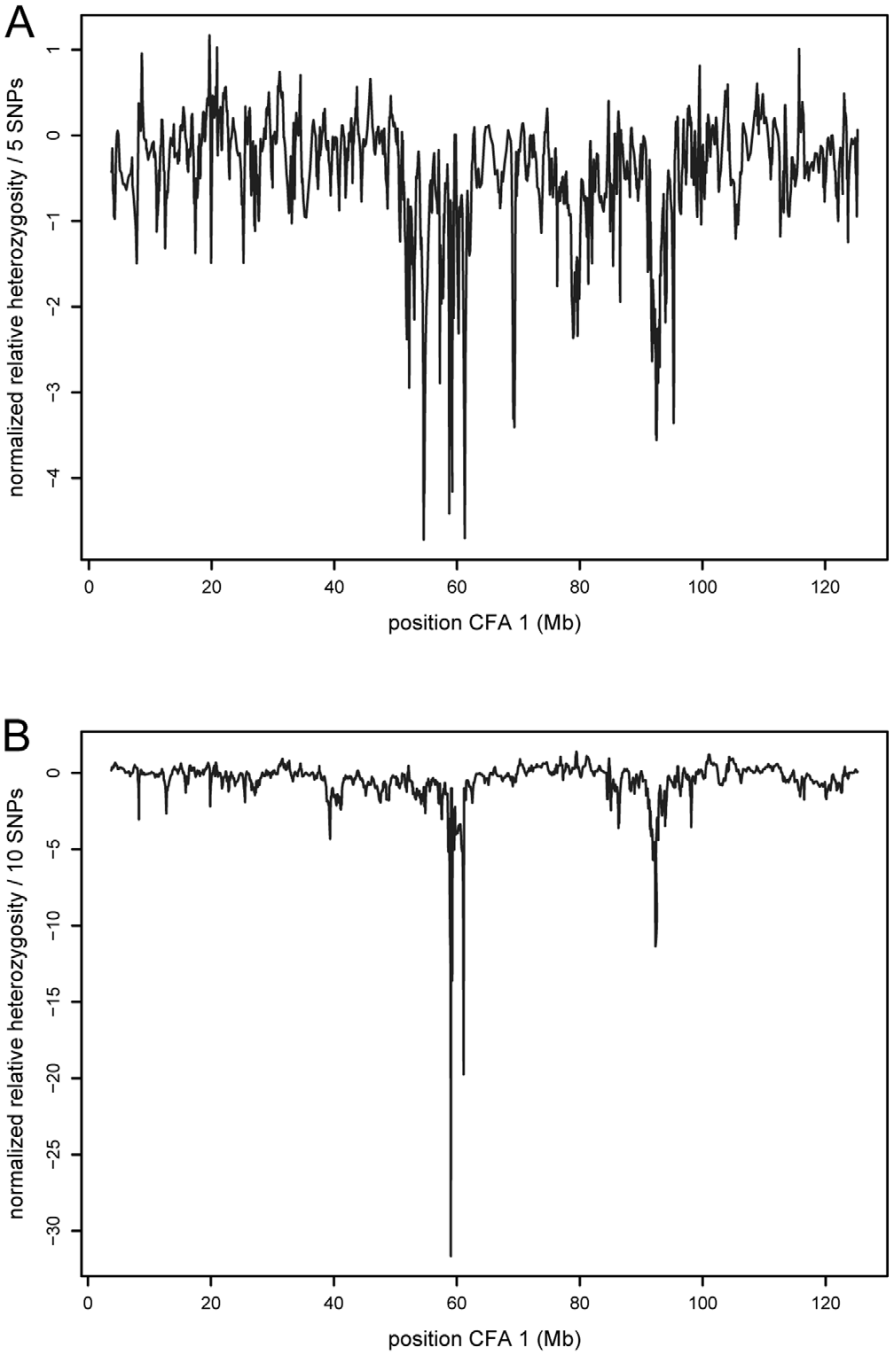
Normalized relative heterozygosity. A. Normalized relative heterozygosity (x axis) is plotted against position on CFA 1 in Mb (y axis) using a five SNP sliding window for brachycephaly in the original dataset. B. Normalized relative heterozygosity (x axis) is plotted against position on CFA 1 in Mb (y axis) using a 10 SNP sliding window for brachycephaly in the second independent dataset.

Both datasets were used to search for selective sweeps by evaluating the normalized relative heterozygosity between affected and unaffected individuals. Both datasets showed dramatic decreases in relative heterozygosity at 59 Mb on CFA 1 for brachycephaly (Figure 4).

**Figure 5:**
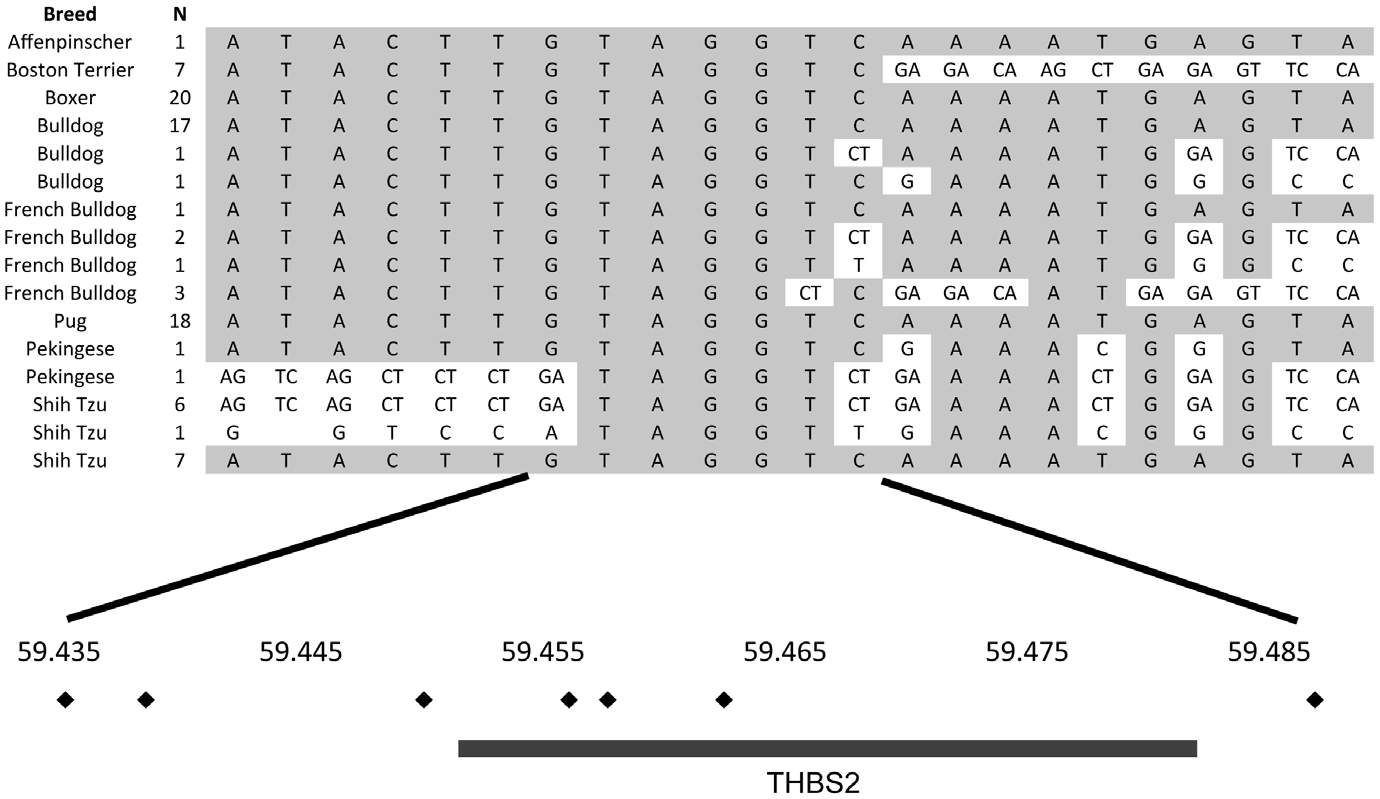
Fine mapping using haplotype analysis for brachycephaly. Haplotypes identified in affected dogs are shown. The breeds (Breed) and number of individuals (N) with each haplotype are shown on the left. In the bottom panel the relative position of the genotyped SNPs (black diamonds) is shown along with the location of the gene (solid bar) in the region.

In order to better define the regions of association, 88 affected dogs and 185 unaffected dogs were genotyped for 49 SNPs spanning the most significantly associated region. A smaller 31 Kb region containing an overlapping homozygous haplotype that contains a single gene, THBS2, was identified within affected breeds (Figure 5). Based on these fine mapping results, the THBS2 gene (59.45–59.47 Mb) and neighboring (58.8–59.0 Mb) SMOC-2 gene (exon 1 from SMOC-2 was unable to be cloned or sequenced) were sequenced in genomic DNA and cDNA from a Boxer (brachycephalic) and a Dalmatian (non-brachycephalic) dog. No sequence changes were detected that were consistent with a causative mutation.

## Discussion

In this study we identified the major locus for brachycephaly using an across breed genome-wide association mapping strategy with only a small number of dogs. Dog breeds are known to have regions of linkage disequilibrium (LD) that are much longer than those seen in humans, thereby allowing trait and disease mapping in dogs with very few individuals and many fewer SNPs than are needed in human studies. However, the length of LD in dogs varies by breed and is in fact highly breed-specific and dependent upon factors such as the age of the breed, population-limiting events such as bottlenecks and popular sire effects, and the breed population size [30]. Across dog breeds, LD is shorter than in human populations, allowing a two stage mapping approach for segregating Mendelian traits [22], [27]. Strong selection for breed defining characteristics along with high inbreeding within dog breeds should cause LD near these loci to be longer. The breeds that were used in this study are predicted to have an average LD of ∼500 Kb based on their population size as compared to breeds in which LD has been measured [24], [31]. Some overlap of this LD allowed for the identification of strongly associated SNPs using an array with ∼50,000 SNPs with an average SNP spacing of 50 Kb. This approach relies upon a high density SNP array in order to identify overlap of regions homozygous for the trait of interest within each breed. A strong signal of selection, as indicated by the decreased relative heterozygosity, allowed for the identification of these regions even though the density of this array was not particularly high. Traits not under strong selection may require even higher density SNP arrays. This is illustrated by canine chondrodysplasia and hyperuricosuria where only single SNPs on this array had significant association with the phenotype.

In all four across breed mapping examples, a *p* value of <0.05 would identify false positive chromosomal locations. In the three cases where the causative mutation had already been identified, the most significant association was properly assigned and was ∼100 times more likely than the next highest association using permutation testing. The falsely decreased p values were more extreme in the second dataset where fewer breeds and more individuals per breed were used. As new arrays are developed that can assay more SNPs, it may be possible to use across breed mapping for more complex traits. This approach is most effective if many breeds are used or if mixed breed animals are used in addition to purebreds to reduce the false positives due to breed differences in allele frequencies.

Across breed mapping, followed by permutation testing and selection of the best locus (>100 more significant than the next highest), successfully identified genomic regions of association for brachycephaly. Fine mapping confirmed and narrowed the interval and two genes, THBS2 and SMOC-2, were implicated. Brachycephaly has been under strong selection as a breed-defining characteristic and was probably fixed either before or soon after the breeds were artificially created. The signatures of selective sweeps [32] within natural populations should be amplified within dog breeds where artificial selection, rather than natural selection, is at work. Using the measure of relative heterozygosity by chromosome helped to define the location of a selective sweep around the brachycephaly locus.

Of the two genes associated with brachycephaly, the most promising candidate gene was THBS2. Thrombospondins (1 and 2) are expressed in bone and cartilage during development and in the adult skeleton [33]. Although null mutations induced in the mouse do not confer a severe skeletal phenotype, they do result in mild brachygnathism in the double mutant; thbs1−/−, thbs2−/− [34]. THBS2 has also been shown to have implications in craniofacial dysmorphism. Mice which overexpress an AP-1 transcription factor, Fra1, have reduced expression of THBS1 and THBS2. During differentiation of osteoblasts, the cells responsible for bone formation, RNA expression of THBS2 dropped over 70% in Fra1 transgenic mice. It was also reduced by over 75% in the long bones of 3-week-old Fra1 mice. The craniofacial dysmorphism presented by the Fra1 transgenic mice was a skull that was shortened along the anterior-posterior axis and larger along the mediolateral axis as compared to wildtype skulls [35].

The second candidate gene for brachycephaly, SMOC2, was located just outside of the critical interval for brachycephalic head type. Nonetheless, disruption of its expression could still be causative of the phenotype. It is a member of the SPARC (secreted protein acidic and rich in cysteine) family, a group of modular, matricellular proteins [36]. They are secreted factors that facilitate interactions between the extracellular matrix of the cell and the surrounding tissue. SMOC2 has a very broad distribution throughout the body and is found in nearly all tissue types [37]. The protein family's namesake, SPARC, also known as osteonectin or BM-40, is expressed primarily during embryogenesis and in adult bone tissue [38]. It has been shown to have roles in angiogenesis, tumorigenesis and wound repair [36]. Due to considerable sequence similarity with SPARC, SMOC2 may have analogous biological functions [39]. Regulatory mutations in either SMOC2 and/or THBS2 could lead to the brachycephalic phenotype. Embryonic expression analysis of these two genes in the developing head of brachycephalic and non-brachycephalic embryos would be necessary to confirm this hypothesis.

As demonstrated by these examples, purebred dogs offer a unique opportunity to identify the genetic etiology of extraordinary phenotypic diversity. Morphological traits such as brachycephaly are characteristic of certain breeds and had a functional purpose at the time that those breeds were created. The fact that such traits were fixed within breeds by strong selection by breeders allows the use of genetic approaches that are not feasible in other species. The small number of samples used for this analysis highlights the power of the canine model system for genetic analysis of traits and implies its potential application to identify other important disease loci in dogs.

## Materials and Methods

Blood samples were acquired from patients of the University of California at Davis William R. Pritchard Veterinary Medical Teaching Hospital. All animals were handled in strict accordance with good animal practice as defined by the relevant national and/or local animal welfare bodies, and all animal work was approved by the Institutional Animal Care and Use Committee (IACUC), approved Protocol for Animal Care and Use #15356. Genomic DNA was isolated using the QiaAmp DNA Blood Mini Kit (Qiagen Inc). DNA samples were purified according to Affymetrix sample preparation guidelines (http://genome.hku.hk/portal/index.php/affymetrix-genechipr-genotyping/sample-preparation) or through the use of Centricon spin columns (Millipore) and eluted in reduced-EDTA TE buffer (10 mM Tris, pH8.0, 0.1 mM EDTA, pH 8.0).

Affymetrix Version 2 Custom Canine SNP arrays were used to obtain genotype calls (Affymetrix). Microarray work was performed by the UC Davis Cancer Center Genomics and Expression Shared Resource, Sacramento, CA. DNA was amplified and labeled according to the manufacturer's protocols, and arrays were washed and stained on a Fluidics Station 450 and were scanned on a GeneChip Scanner 3000. A complete list of the dogs used is available in supplemental tables S1 and S2 for the two datasets.

Only genotype calls with a *p* value of <0.01 were used for analysis. The program PLINK [40] was used for the genome-wide association analysis using the v2 platinum SNP set with minor allele frequencies >0.05, 75% genotype calls and no more than 25% missing genotypes per individual. Permutation testing for whole genome association with 50,000 permutations was performed using PLINK. Haplotype and association analysis for single chromosomes was performed using the program Haploview [41].

The second independent dataset was also run on the Affymetrix Version 2 Canine array with 49,663 SNPs (50K). Approximately 10 dogs from each of the breeds used were randomly chosen for analysis. For brachycephaly, three breeds were considered as cases (Pugs, Boxer and Pekingese) and 13 breeds (Afghan Hound, Akita, Basenji, Basset, Dachshund, Eurasier, German,Short-Haired,Pointer, Greyhound, Leonberger, Pembroke Welsh Corgi, Poodle, Saluki, and TibetanTerrier) were considered as controls. Genome wide association calculations for single marker chi-square were performed using the software package PLINK [40].

To check for a selective sweep, a relative decrease in heterozygosity was evaluated. When determining results for a decrease in heterozygosity relative to controls, all dogs with a call rate of >75% were used. SNPs with a call rate < 95% were removed. The rate of heterozygosity was first calculated for control breeds and case breeds separately and then the ratio between those was used as a measure of relative heterozygosity. The relative heterozygosity was normalized in a way that gives negative values when cases are less heterozygous than controls and otherwise positive values. The normalization was performed by the following formula: ratio = 1 -1/ratio if ratio < 1 and ratio ≠ 0. In other cases, the ratio was diminished by 1.

SNPs chosen for fine-structure mapping were harvested through the online Entrez SNP database, dbSNP (http://www.ncbi.nlm.nih.gov/SNP/). SNPs were selected for each region based on known polymorphisms between the Boxer reference sequence and other breeds. Acceptable, documented SNPs were interrogated in 96 affected dogs from various breeds and 187 controls. These samples were genotyped for the selected SNPs by Sequenom-based MassARRAY genotyping through GeneSeek (Lincoln, NE).

Primer pairs for the putative 5′ and 3′ UTRs, the predicted exons in genomic DNA and cDNA and the predicted intron-exon boundaries were created based on the canine UCSC reference Boxer sequence. Primers for the chromosomal regions containing the candidate genes were designed through the Primer3 website (http://frodo.wi.mit.edu/cgi-bin/primer3/primer3_www.cgi) (Table S3). The designed primers were used for PCR and sequencing.

RNA was extracted from liver samples from a Boxer and a Dalmatian for brachycephalic head type using the Fast Track 2.0 mRNA Isolation Kit (Invitrogen). cDNA was synthesized from these samples using the SuperScript III First-Strand Synthesis System (Invitrogen). RT-PCR was performed for both THBS2 (liver) and SMOC2 (liver) in order to define the cDNA structure of these genes.

PCR was performed on a GeneAmp PCR system 9700 (Applied Biosystems) with 1 µL DNA, 2.5 mM dNTP, 0.5U AmpliTaq Gold Polymerase (Applied Biosystems), 1 µL 10X Buffer II with 1.5 mM MgCl2, and water to 20 µl. Amplification parameters were: 95°C for 12 min, 35 cycles of 94°C for 30 s, primer-specific annealing temperature for 30 s, and 72°C for 45 s, with a final extension of 72°C for 20 min. Products were purified using the QIAquick PCR Purification Kit (Qiagen Inc).

Purified PCR products were sequenced with the ABI Prism BIGDye Terminator Cycle Sequencing Kit (version 3.1) and unincorporated dye terminators were removed with the BigDye XTerminator Purification kit (Applied Biosystems). Sequencing products were processed on an ABI Prism 3100 Genetic Analyzer (Applied Biosystems) and visualized using Chromas2 (Technelysium). Sequences were obtained from a Boxer and a Dalmatian control for brachycephalic head type. The available canine genome sequence (Boxer) was used as an additional affected dog for brachycephalic head type. Sequences were submitted to GenBank (accession numbers∶GU123606-GU123608). Sequence alignment was performed using VectorNTI software (Informax).

## Supporting Information

Table S1 (0.02 MB XLS)

Table S2 (0.03 MB XLS)

Table S3 (0.03 MB XLS)
